# A Nationwide Survey of the Spine Education of Medical Students

**DOI:** 10.7759/cureus.106443

**Published:** 2026-04-04

**Authors:** Matthew C Henn, Ali Rae, Adeline L Fecker, Matthew McIntyre, Amalia Larsen, James Wright

**Affiliations:** 1 Neurological Surgery, Oregon Health and Science University School of Medicine, Portland, USA; 2 Neurological Surgery, Oregon Health and Science University, Portland, USA

**Keywords:** medical education, medical education survey, medical student education, neurosurgery education, orthopedic education, spine, spine curriculum, spine education

## Abstract

Introduction

Spinal disease is prevalent, yet education regarding these disorders in undergraduate medical education is limited.

Objective

This study assesses medical students' exposure, confidence, competence, and attitudes regarding their spine anatomy and pathology curricula.

Methods

US allopathic and osteopathic medical students completed an anonymous, uncompensated survey, including 17 questions on attitudes towards spine education and 15 on spine anatomy and pathology. Surveys completed in <180 seconds or <80% complete were excluded. Students estimated their hours of spine education and rated their comfort in diagnosing spine pathology. Data was analyzed using Stata (StataCorp, College Station, TX, USA) and RStudio (Posit PBC, Boston, MA, USA).

Results

Of 491 survey respondents, 252 (51.2% allopathic, 48.8% osteopathic) met the inclusion criteria. Surgery and medicine were the most common specialty interests. Students scored significantly higher in lumbar competence (2.20 vs. 1.96; p<0.001, F = 7.40) and confidence (1.72 vs. 0.82; p<0.001, F = 38.66) questions than in cervical spine. Students who reported over 20 hours of spine anatomy teaching answered more lumbar spine questions correctly and reported more confidence answering both cervical and lumbar spine questions. Students with reported interest in either neurosurgery or orthopedics had a non-significant trend to answer more questions correctly overall but were significantly more confident with cervical spine questions (p=0.02, x^2^ = 19.58, df = 4). A majority (59.1%) of participants wished for more didactic time on spine pathology, and 39.5% of participants expressed dissatisfaction with clinical rotation didactics.

Conclusions

US medical students demonstrated disparity in recognizing cervical versus lumbar spine pathologies. Most respondents desired more spine-focused didactics, suggesting an area of improvement in medical education by balancing spine education to reflect disease prevalence.

## Introduction

Spinal diseases comprise a large portion of clinical practice across many specialties, including primary care, emergency medicine, neurology, and several surgical subspecialties. Back pain is a common complaint encountered by primary care and emergency medicine providers, with a 2022 meta-analysis revealing that 36.1% of older adults have a 12-month prevalence of low back pain, including many pathologies [1]. Unfortunately, many patients with spinal pathologies experience considerable diagnostic delays, and early intervention can prevent irreversible damage [2-4]. Only 35.0% of physicians in first-line provider specialties reported being ‘very prepared’ to handle spinal emergencies, specifically cervical myelopathy, epidural abscess, and lumbar radiculopathy [5]. Despite their prevalence in clinical practice, diseases of the spine are likely underrepresented in undergraduate medical education (UME) curricula. A recent study in the United Kingdom (UK) found that degenerative cervical myelopathy (DCM) appears to be an overlooked condition in medical education, with 72% of surveyed students reporting no instruction on DCM, and nearly half rating their knowledge of this condition as ‘terrible’ [6]. Another UK study revealed that students struggled with their knowledge of DCM management [7].

It is well documented that medical student education of the musculoskeletal system is lacking. An institutional survey conducted at Johns Hopkins School of Medicine demonstrated that only 19% of medical students passed a validated orthopedic examination of musculoskeletal competency [8]. However, students who participated in an elective musculoskeletal medicine course demonstrated improved performance, with 68% passing the exam. This improvement was accompanied by increasing diagnostic and treatment confidence as students advanced through medical school. Similarly, a separate study examining medical students’ confidence in their musculoskeletal knowledge revealed unsatisfactory levels of knowledge and confidence, both of which improved after a focused didactic [9]. At Harvard Medical School, a study of 249 students showed similar gaps in knowledge and confidence, and identified the musculoskeletal system as a potential curriculum gap [10]. Additionally, a survey in the UK highlighted deficiencies in orthopedic and trauma education, with medical schools failing to adequately prepare their graduates in these areas [11].

Despite the documented gaps in broad musculoskeletal education, there is a notable lack of published literature specifically addressing medical students’ confidence and competence regarding the spine. This study aims to survey the current state of spine education and diagnostic competency among United States (US) allopathic and osteopathic medical students. We hypothesize that the majority of students surveyed will not demonstrate competence and confidence in their levels of cervical and lumbar spine knowledge.

## Materials and methods

Recruitment methods

This study institutional review board protocol (STUDY00026701) was approved by the author’s institution in accordance with guidelines. Subjects were recruited via email containing a link to an anonymous uncompensated Qualtrics (Provo, UT, USA) survey. One hundred fifty-seven allopathic and osteopathic schools were contacted directly, with the remaining schools unable to be included due to no response or declination. Most schools declined sending out the survey or did not respond. When approved, medical students were contacted via medical administrators and student groups. The survey recruitment period occurred between March 5, 2024, and May 20, 2024. Informed consents (Consent to Participate and Consent to Publish) were obtained from all participants.

Inclusion and exclusion criteria

Only US-accredited allopathic or osteopathic school medical students were included in the survey. Research or gap year students were also included. Surveys were untimed, but those completed in <180 seconds were excluded to minimize random data. Surveys <80% complete were also excluded for completeness.

Survey development

The survey was developed by the authors of this manuscript and included six background and demographic questions. There were 17 questions on attitudes towards spine education on a Likert scale, six questions on spine anatomy, four ‘board-style’ questions testing spinal disease recognition, two questions specific to cervical spondylotic myelopathy, two questions on central cord syndrome, one question regarding lumbar spondylolisthesis, and one question on cauda equina syndrome (CES). ‘Board-style’ questions included a full clinical vignette. It was reviewed to ensure appropriate content, legibility, and understanding by residents and faculty of the author’s neurosurgery department.

Data collection

Responses were collected and stored in the Qualtrics database and subsequently extracted to Microsoft Excel (Redmond, WA, USA) on a secure server.

Competence and confidence

From the 15 anatomy and pathology multiple choice questions, of which there were seven cervical spine, five lumbar spine, and three general questions, competence was analyzed based on the number of correctly answered questions: less than four correct labeled as ‘not competent’, four to seven correct as ‘moderately competent’, and eight to 10 correct as ‘highly competent.’ Confidence levels were assessed on a scale between zero and four, with points awarded for ‘somewhat agree’ or ‘strongly agree’ responses to confidence questions.

Data analysis

This study employed within-subject analysis with chi-square test, paired t-test, z-tests, and ANOVA tests as appropriate. Statistical significance was set as a two-tailed p-value <0.05. Knowledge questions that were left blank were considered incorrect. The statistical analysis was performed utilizing Stata (StataCorp, College Station, TX, USA) and RStudio (Posit PBC, Boston, MA, USA) [12,13]. Tables and figures were made using Microsoft Excel and Word.

## Results

Demographics

**Figure 1:**
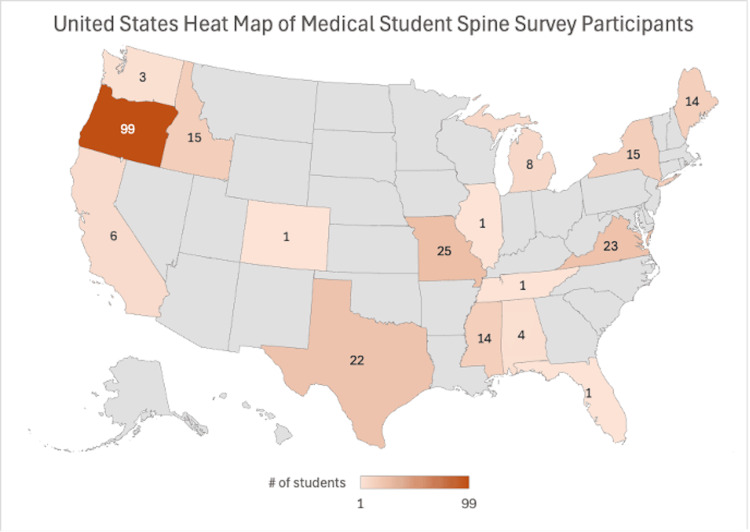
United States Heat Map of Medical Student Spine Survey Participants

A total of 491 students responded to the survey, with 242 students from 27 medical schools fully completing it and an additional 10 students completing at least 80%. One hundred twenty-nine (51.2%) current allopathic and 123 (48.8%) osteopathic students were included. Students from various medical schools were represented (Figure 1). Eighty-two students (32.5%) identified as first-year medical students, 77 (30.6%) as second-year, 40 (15.9%) as third-year, 46 (18.3%) as fourth-year, and seven (2.8%) as research-year or gap-year students. The most common specialties students planned on applying into were surgery, including subspecialties (58 students; 23.0%), a specialty of medicine (44 students; 17.5%), and family medicine (36 students; 14.3%). Ten students (4.0%) planned to apply into neurological surgery, and 20 students (7.9%) were interested in orthopedic surgery (Table 1).

**Table 1:** Characteristics of Included Participants

Variable	Category	Number of Students N(%)
Total Students	All categories	252
Type of School	Allopathic	129 (51.2%)
	Osteopathic	123 (48.8%)
Class Year	M1	82 (32.5%)
	M2	77 (30.6%)
	M3	40 (15.9%)
	M4	46 (18.3%)
	Research/Year off	7 (2.8%)
Specialty interest	Medicine (specialty)	44 (17.5%)
	Family Medicine	36 (14.3%)
	Emergency Medicine	31 (12.3%)
	Surgery (other)	28 (11.1%)
	Internal Medicine	28 (11.1%)
	Pediatrics	24 (9.5%)
	Obstetrics and Gynecology	20 (7.9%)
	Orthopedic Surgery	20 (7.9%)
	Neurological Surgery	10 (4.0%)
	Radiology	7 (2.8%)
	Not listed	4 (1.6%)

Cervical spine

To assess cervical spine knowledge, central cord syndrome and cervical spondylotic myelopathy were tested. Based on survey results, second-year (p<0.020, F = 8.93), third-year (p=0.001, F = 8.93), and fourth-year students (p<0.03, F* *= 8.93) were significantly more competent than first-year students. Sixty students (23.8%) indicated that they ‘somewhat’ or ‘strongly’ agreed with feeling comfortable diagnosing cervical myelopathy, while only 25 (9.9%) reported confidence in diagnosing central cord syndrome. One hundred thirty-seven students (54.4%) reported confidence level zero. Forty-two students (16.7%) were not competent and not confident in cervical spine-related questions. Thirty-four students (13.5%) were highly competent, with 10 of these students (29.4%) still reporting low confidence (confidence level zero) (Table 2). Third-year (p<0.002, F = 18.21) and fourth-year medical students (p<0.001, F = 18.21) were significantly more confident than first-year medical students.

**Table 2:** Student Competence and Confidence in Spine Pathology

Spine Region	Metric	Category	Number of Students N(%)
Cervical Spine	Competence	Not competent (0-3 correct)	44 (17.5%)
		Moderately competent (4-7 correct)	174 (69.1%)
		Highly competent (8-10 correct)	34 (13.5%)
	Confidence Level	0	137 (54.4%)
		1	59 (23.4%)
		2	35 (13.9%)
		3	7 (2.8%)
		4	14 (5.6%)
Lumbar Spine	Competence	Not competent (0-3 correct)	29 (11.5%)
		Moderately competent (4-7 correct)	144 (57.1%)
		Highly competent (8-10 correct)	79 (31.4%)
	Confidence Level	0	69 (27.4%)
		1	43 (17.1%)
		2	68 (27.0%)
		3	33 (13.1%)

Lumbar spine

To assess lumbar spine knowledge, lumbar spondylolisthesis and CES were tested. Fourth-year medical students were significantly more competent with lumbar spine knowledge compared to first-year (p=0.002, F = 8.93) and second-year students (p=0.003, F = 8.93). Seventy-nine (31.4%) students were highly competent. Sixty-nine students (27.4%) selected confidence level zero, with more than half of students (140; 55.6%) answering questions with at least confidence level two. Notably, 68% of students indicated that they ‘somewhat’ or ‘strongly’ agreed with feeling comfortable diagnosing CES. Five students (6.3%) reported low confidence (confidence level zero) (Table 2). Third-year (p<0.0001, F = 18.21) and fourth-year medical students (p<0.0001, F = 18.21) were significantly more confident than first-year medical students. They were also significantly more confident than second-year students (p=0.007; p<0.0001, F = 18.21).

Cervical and lumbar spine comparison

**Figure 2:**
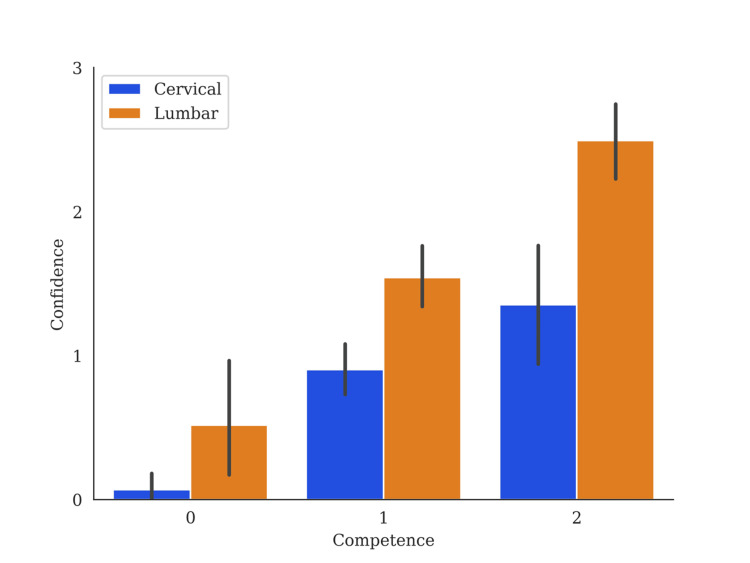
Competence Against Confidence in Medical Students Spine Knowledge Bar graphs represent mean confidence levels with standard error of the mean at each competence level. Competence level zero is less than four questions correct, competence level one is between four and seven correct, and competence level two is more than eight correct.

Students were more competent answering lumbar spine-related questions when compared to cervical spine questions, with competence scores of 2.20 and 1.96, respectively (p<0.01, F = 7.40). Students reported feeling more confident answering lumbar-related spine questions, with a mean confidence score of 1.72 for lumbar and 0.82 for cervical spine questions (p<0.01, F = 38.66). A comparison of confidence versus competence can be found in Figure 2. Students interested in spine surgery fields, including orthopedic surgery and neurosurgery, answered more cervical (p=0.36, x^2^ = 19.58, df = 4) and lumbar (p=0.10, x^2^ = 19.58, df = 4) questions correctly, however this was not statistically significant. They were also more confident than other students with cervical spine questions (p=0.02, x^2^ = 19.58, df = 4).

Anatomy instruction

Ninety-one students (36.1%) estimated they had over 20 hours of spine anatomy education to date, including both pre-clerkship and clerkship years. Those who reported over 20 hours of spine anatomy answered more lumbar spine questions correctly than those who reported <20 hours (5.85 compared to 5.39). Students with over 20 hours of spine anatomy also reported more confidence answering both cervical and lumbar spine questions, with reported confidence levels of 1.10 and 2.2 compared to 0.66 and 1.45, respectively. First-year students (36.6%) had a lower percentage of students reporting >20 hours of spine anatomy education compared to third-year (40.0%) and fourth-year students (54.3%).

Satisfaction

The majority of students (158; 65.3%) were satisfied with their level of spine education in anatomy class while 54 students (22.3%) were dissatisfied. Regarding pre-clinical didactics, 134 (55.4%) were satisfied with the amount of spine education and 63 students (26.0%) were dissatisfied. In clinical rotations, 37 students (33.9%) were satisfied with their breadth of spine education, and 43 students (39.5%) were dissatisfied. Ninety-three students (38.4%) wished for more educational time on spinal anatomy during didactics. Furthermore, 143 students (59.1%) wished there was more educational time spent during didactics on spinal disease pathology.

## Discussion

This nationwide survey revealed significant disparities between medical students’ competence and confidence levels in cervical versus lumbar spine knowledge. Although 69.1% of students demonstrated moderate competence in cervical spine knowledge, the majority (54.4%), including a mix of student years with no significance found in any particular year, reported low confidence in diagnosing and treating cervical spine pathology. This lack of confidence, even among students with high competence, highlights a potential gap in medical education, where theoretical knowledge may not translate into clinical confidence. The two cervical spine pathologies tested, central cord syndrome and cervical spondylotic myelopathy, represent 9.0% and 7.5% of adult spinal cord injuries, respectively [14,15]. The commonality of these pathologies indicates they are important to recognize and feel comfortable diagnosing. The survey also showed that students performed better on the lumbar spine questions, which may reflect the emphasis of CES on medical student examinations [16].

Repeated exposure has been found to positively correlate with confidence in medical decision-making [17]. This is demonstrated in our study by the significantly higher confidence scores in both lumbar and cervical spine-related questions with students who reported over 20 hours of spine anatomy. While the additional time spent may represent personal interest, it may also include elective didactic education outside of required didactics. In the UK, the majority of medical schools utilize gait-arms-legs-spine (GALS) as an educational resource to teach functional movements and screening tools, and 90% of medical students who were surveyed following this training reported improved confidence in their musculoskeletal examination [18]. A separate study in Louisiana developed a six-week course that included lectures, anatomy, and small-group sessions on musculoskeletal medicine, and the average student scored almost 20% higher on competency exams following the course, with a majority reporting high satisfaction rates [19].

These proposed interventions require time and resources to execute. One study showed that while neurosurgeons are the most prominent sources of information for head and spine injuries, they are not significantly involved in medical student education regarding common neurosurgical issues [20]. However, spine surgeons do not need to be the sole educators of spine pathology at the UME level. A recent pilot study in the UK utilized social media as a medium to host a series of case-based discussions regarding common neurosurgical conditions in practice [21]. Participants in these case-based discussions reported an increase of 77% in their knowledge levels afterwards. Additionally, all participants noted they would attend future sessions.

Our findings also revealed mixed satisfaction levels with spine education amongst medical students. While a majority were satisfied with their dedicated anatomy class (65.3%) and pre-clinical didactic education of the spine (55.4%), there was also notable dissatisfaction with spine education during clinical rotations (39.5%). Furthermore, a significant number of students expressed a desire for more educational time on spinal anatomy (38.4%) and spinal disease pathology (59.1%) during didactics. This suggests that while the foundational knowledge in spinal disorders is adequately covered, practical application during clinical rotations may be lacking. These findings are consistent with a study done in the UK surveying medical students’ exposure and satisfaction to trauma care during their clinical phase of schooling, where the majority of students believed their trauma medical training to be inadequate [22]. A reassessment of how spine education is integrated into clinical training, particularly emphasizing increased opportunities for hands-on and case-based learning, might be beneficial.

Future directions

Students who reported more hours of spine education and students with reported interest in spine surgery demonstrated higher competence and confidence scores on our survey than those with fewer hours of exposure. This may be due to additional extracurricular exposure to spine pathology or varying degrees of curricular emphasis. A potential intervention would be a dedicated spine clinic, spine surgery experience, or supplementary curricular teaching during student’s clerkship rotations during their clinical years. Research indicates that even small curriculum adjustments can significantly impact learning [23]. In one study, the introduction of a seven-lecture series on neurology, neurosurgery, and rehabilitation medicine topics significantly improved third-year medical students’ performance on exams testing the recognition and management of degenerative spine disease, spine injury, stroke, and other common neurosurgical disorders [23]. An alternative approach could involve augmenting case-based learning and simulation experiences into clinical education curricula, as evidence suggests that case-based learning is more effective than didactic lectures [24,25]. Furthermore, the higher competence and confidence levels in lumbar spine knowledge suggest that more emphasis could be placed on cervical spine education during medical undergraduate years to yield comparable outcomes.

Limitations

Like all such studies, our analysis is limited by survey response design. Self-reported data is subject to response bias, and the cross-sectional design provides a snapshot of student competence and confidence at a single point in time, which may not capture changes during their medical education. Furthermore, the competence-related survey questions were limited in breadth, with only a few important pathologies and anatomy tested, and they were geared specifically towards the cervical and lumbar spine, leaving out many other spinal pathologies and anatomy. Given that this study was done towards the end of the academic year, results could also skew in favor of students having more knowledge than they would earlier in the year.

We also do not know whether students accessed outside resources to take the survey, as the nature of the survey being an anonymous link meant that students could take it privately. Additionally, the distribution of students skews towards Oregon medical schools. Though 157 medical institutions were contacted, only 27 schools both accepted to send the survey out and had students fill the survey out with appropriate inclusion criteria. This challenged the study from producing substantially more completed surveys and limits generalizability. Unfortunately, many students who were excluded due to survey completion percentage ended the exam once the longer ‘board-style’ questions were presented. However, the full survey lasted 10-15 minutes and adding additional topics would have lengthened it more, perpetuating our loss of complete surveys. Finally, knowledge gained over the four years could also play into the results found. Future longitudinal studies could track the progression of competence and confidence in spinal anatomy and pathology throughout medical school.

## Conclusions

This study reveals disparity among US medical students in recognizing cervical spine pathologies compared to lumbar spine pathologies. Additionally, the majority of respondents wished they spent more time learning spine pathology in didactics. These findings reveal a potential area of improvement in current medical education by balancing spine education to reflect disease prevalence in our patient population. 

## Appendices

Spine Survey

Demographics

1.     What is your school name?

Drop-down menu

2.     What year are you in school?

M1

M2

M3

M4

Research/Year Off

3.     Estimate how many hours of spine anatomy education you’ve had to date

< 5

5 - 10

11 - 20

20+

4.     Estimate how many hours of cardiac anatomy education you’ve had to date

< 5

5 - 10

11 - 20

20+

5.     Estimate how many hours of abdominal cavity anatomy education you’ve had to date

< 5

5 - 10

11 - 20

20+

6.     What specialty do you tentatively plan on applying into?

Drop-down menu

Anatomy

7.     Please choose the correct order of these spinal ligaments from anterior to posterior: ligamentum flavum (LF), anterior longitudinal ligament (ALL), supraspinous ligament (SSL), posterior longitudinal ligament (PLL), and interspinous ligament (ISL).

ALL, PLL, ISL, LF, SSL

ALL, PLL, LF, ISL, SSL

SSL, ISL, LF, PLL, ALL

SSL, LF, ISL, PLL, ALL

8.     When performing a lumbar puncture, the iliac crest approximates what spinal level 

L1-2

L2-3

L3-4

L4-5

L5-S1 

9.     Match the correct answer: The nerve root of C5 exits ____ the C5 vertebral body; The T5 nerve root exits ___ the T5 vertebral body. 

Above; above

Above; below

Below; below

Below; above

10.  A traumatic fracture of a vertebral body with height loss and retropulsion into the spinal canal implies what type of fracture? 

Burst 

Compression

Translation

Pathologic 

Jefferson 

11.  The most sensitive and specific imaging test to rule out a cervical spine fracture is what?

MRI

CT

X-ray

12.  What muscle would you expect to be atrophied in a C5 radiculopathy?

Deltoid 

Triceps

Opponens pollicis 

Iliopsoas 

Spinal Diseases - Objective

13.  A 67-year-old male presents to the clinic with tingling and weakness in his left shoulder. He reports inability to work and perform fine motor movements such as signing his name. He is often tripping and falling at home and has difficulty buttoning his shirt. An MRI of his spine shows moderate to severe C5, C6, and C7 disc herniation causing cord compression with cord signal of the spinal cord at these segments. What is the most likely diagnosis?

Multiple sclerosis 

Cervical spondylotic myelopathy

Amyotropic lateral sclerosis

Cervical radiculopathy 

Cauda equina syndrome 

14.  A 46-year-old female presents to the clinic with a loss of sensation in her C4-T2 dermatomes after a fall in the bathroom striking her forehead on the sink. She reports challenges with motor movements as well, making it hard to go about her daily tasks including using the bathroom. An MRI of her spine shows a syrinx in the spinal canal between segments C4-C7. What is the most likely clinical diagnosis?

Central cord syndrome

Anterior cord syndrome

Brown Sequard syndrome

Transverse cord syndrome

15.  A 65-year-old male presents to the clinic with reports of low back pain, with onset about three months ago after he fell down his stairs at home. He mentions that his pain worsens when bending over and he has difficulty standing or walking for long periods of time. During a physical exam, reduced sensation and tingling was found in his right foot. An x-ray of his spine shows an anterior slip of the L5 vertebral body on S1. What is the most likely cause of his symptoms?

Lumbar spondylolisthesis

Ankylosing spondylitis

Sacral spondylosis

Compression fracture 

16.  A 25-year-old female acrobat presents to the clinic due to loss of bladder and anal sphincter control that started 12 hours ago after a hard fall in practice. On physical exam, the patient exhibits saddle anesthesia, a 500mL post void residual, and 2/5 strength in her hip flexion, knee extension, dorsiflexion, and plantarflexion bilaterally as well as absent knee and ankle reflexes. An MRI of her spine indicates a severely herniated disc at L2. What is the most likely diagnosis?

Lumbar radiculopathy 

Cauda Equina syndrome

Syringomyelia

Tabes dorsalis

Spinal Diseases - Subjective

Cervical spondylotic myelopathy

17.  I would feel comfortable diagnosing cervical spondylotic myelopathy

Strongly disagree

Slightly disagree

Neither agree nor disagree

Slightly agree

Strongly agree

18.  I would feel comfortable treating cervical spondylotic myelopathy

Strongly disagree

Slightly disagree

Neither agree nor disagree

Slightly agree

Strongly agree

19.  Cervical spondylotic myelopathy is an emergency

Strongly disagree

Slightly disagree

Neither agree nor disagree

Slightly agree

Strongly agree

20.  Cervical myelopathy is a condition that refers to ____ of the cervical spinal cord. 

Destruction

Compression

Claudication

Expansion

Central Cord Syndrome

21.  I would feel comfortable diagnosing central cord syndrome

Strongly disagree

Slightly disagree

Neither agree nor disagree

Slightly agree

Strongly agree

22.  I would feel comfortable treating central cord syndrome

Strongly disagree

Slightly disagree

Neither agree nor disagree

Slightly agree

Strongly agree

23.  Central cord syndrome is an emergency

Strongly disagree

Slightly disagree

Neither agree nor disagree

Slightly agree

Strongly agree

24.  Which physical exam findings might you find in a patient presenting with central cord syndrome?

Bilateral loss of pain and sensory sensation

Loss of bladder control

Absent knee reflexes

Ataxic gait

25.  Central cord syndrome is a condition that is closely related to what disease process?

Tapes dorsalis

Syringomyelia

Vitamin B12 deficiency

Amyotrophic lateral sclerosis (ALS)

Lumbar Spondylolisthesis

26.  I would feel comfortable diagnosing lumbar spondylolisthesis

Strongly disagree

Slightly disagree

Neither agree nor disagree

Slightly agree

Strongly agree

27.  I would feel comfortable treating lumbar spondylolisthesis

Strongly disagree

Slightly disagree

Neither agree nor disagree

Slightly agree

Strongly agree

28.  Lumbar spondylolisthesis is an emergency

Strongly disagree

Slightly disagree

Neither agree nor disagree

Slightly agree

Strongly agree

29.  What is the most common spinal cord location for degenerative lumbar spondylolisthesis?

L1-L2

L2-L3

L3-L4

L4-L5

L5-S1

Cauda Equina Syndrome

30.  I would feel comfortable diagnosing cauda equina syndrome

Strongly disagree

Slightly disagree

Neither agree nor disagree

Slightly agree

Strongly agree

31.  I would feel comfortable treating cauda equina syndrome

Strongly disagree

Slightly disagree

Neither agree nor disagree

Slightly agree

Strongly agree

32.  Cauda equina syndrome is an emergency

Strongly disagree

Slightly disagree

Neither agree nor disagree

Slightly agree

Strongly agree

33.  Which of the following presentations is most commonly found in cauda equina syndrome?

Inability to walk

“Saddle anesthesia” sensory disturbance

Sexual dysfunction

Sciatica (pain in the back/and or legs)

End of Survey Questions

34.  I am satisfied with my level of spine education in anatomy class

Strongly disagree

Slightly disagree

Neither agree nor disagree

Slightly agree

Strongly agree

35.  I am satisfied with my level of spine education in didactics

Strongly disagree

Slightly disagree

Neither agree nor disagree

Slightly agree

Strongly agree

36.  I am satisfied with my level of spine education in clinical rotations

Strongly disagree

Slightly disagree

Neither agree nor disagree

Slightly agree

Strongly agree

Not applicable

37.  I wish there were more educational time spent during didactics on spinal anatomy

Strongly disagree

Slightly disagree

Neither agree nor disagree

Slightly agree

Strongly agree

38.  I wish there were more educational time spent during didactics on spinal diseases

Strongly disagree

Slightly disagree

Neither agree nor disagree

Slightly agree

Strongly agree
